# *Litopenaeus vannamei* hemocyanin exhibits antitumor activity in S180 mouse model *in vivo*

**DOI:** 10.1371/journal.pone.0183783

**Published:** 2017-08-30

**Authors:** Shangjie Liu, Liyuan Zheng, Jude Juventus Aweya, Zhou Zheng, Mingqi Zhong, Jiehui Chen, Fan Wang, Yueling Zhang

**Affiliations:** 1 Department of Biology and Guangdong Provincial Key Laboratory of Marine Biotechnology, Shantou University, Shantou, China; 2 Reproductive Medicine Department, Affiliated Jingzhou Central Hospital of Tongji Medical College, Jingzhou, China; Columbia University, UNITED STATES

## Abstract

Hemocyanin is a multifunctional glycoprotein, which also plays multiple roles in immune defense. While it has been demonstrated that hemocyanin from some mollusks can induce potent immune response and is therefore undergoing clinical trials to be used in anti-tumor immunotherapy, little is currently known about how hemocyanin from arthropods affect tumors. In this study we investigated the anti-tumor activity of hemocyanin from *Litopenaeus vannamei* on Sarcoma-180 (S180) tumor-bearing mice model. Eight days treatment with 4mg/kg bodyweight of hemocyanin significantly inhibited the growth of S180 up to 49% as compared to untreated. Similarly, histopathology analysis showed a significant decrease in tumor cell number and density in the tissues of treated mice. Moreover, there was a significant increase in immune organs index, lymphocyte proliferation, NK cell cytotoxic activity and serum TNF-α level, suggesting that hemocyanin could improve the immunity of the S180 tumor-bearing mice. Additionally, there was a significant increase in superoxide dismutase (SOD) activity and a decrease in the level of malondialdehyde (MDA) in serum and liver, which further suggest that hemocyanin improved the anti-oxidant ability of the S180 tumor-bearing mice. Collectively, our data demonstrated that *L*. *vannamei* hemocyanin had a significant antitumor activity in mice.

## Introduction

Hemocyanin is a large copper containing glycoprotein which functions as a respiratory molecule in mollusk and arthropods. Recent studies revealed that hemocyanin played additional roles in energy storage, osmoregulation, molting regulation, and non-specific immune defense [1–6]. In 1998, Decker *et al*. first reported that hemocyanin from the spider *eurypelma californicum* had phenoloxidase activity [7]. Following this, other researchers documented that hemocyanin from *Limulus polyphemus*, *Oncomelania hupensis*, and *Cherax quadricarinatus* could also be functionally converted into phenoloxidase-like enzymes after treatment with sodium dodecyl sulfate (SDS) and phosphatidylserine [8–11]. In addition, it has been reported that hemocyanin from *Penaeus monodon* possessed antiviral activities against a variety of viruses including DNA and RNA viruses [12]. Similarly, Nesterova *et al*. and Zagorodnya *et al*. reported that *Rapana venosa* and *Helix Lucorum* hemocyanin could act as an antiviral agent against Epstein-Barr virus (EBV) [13,14]. Besides this, Jiang *et al*. found that hemocyanin from horseshoe crab *Tachypleus tridentatus*, when treated with exogenous protease, could exhibit a strong antimicrobial defense by reactive oxygen species (ROS) production [15]. Destoumieux-Garzón reported that the C-terminal fragment of hemocyanin from penaeid shrimps *Penaeus vannamei* and *Penaeus stylirostris* had broad antifungal activities [16]. Additionally, our previous evidences indicated that hemocyanin from shrimp *L*.*vannamei* and mud crab *Scylla serrata* could bind with some bacteria including *Vibrio parahaemolyticus*, *Aeromona hydrophila*, and *Escherichia coli* K12, as an agglutinin. Apart from this, our previous studies unveiled that hemocyanin could react with anti-human Ig as an antigen, bind with erythrocytes as a hemolysin, and enhance shrimp's immune response as an immune-enhancement protein [17–20]. More recently, a novel 18.4 kDa hemocyanin fragment was identified in *V*. *parahaemolyticus* challenged *L*. *vannamei* sera which had some immunological functions both *in vitro* and *in vivo* [21].

Several research findings have shown that hemocyanins from some mollusks had antitumor effects. In 1974, Olsson *et al*. immunized patients with *keyhole limpet* hemocyanin (KLH) from *Megathura crenulata* and found a marked reduction in the recurrence of superficial bladder cancer [22]. After that, researchers found KLH to possess growth inhibition effects on multiple cancer cell lines including breast (ZR75-1 and MCF-7) and pancreas (PANC-1) [23]. Also, hemocyanins from *Concholepas concholepas* (CCH) and *Fissurella latimarginata* (FLH) have been demonstrated to possess anti-tumor activity [24, 25]. Of which, CCH has been further used as a carrier [26], an antigen [27], and an adjuvant in dendritic cell (DC) immunotherapy of patients with prostate cancer [28].

Mollusk hemocyanins are enormous glycoproteins (4 to 8 MDa) formed by a complex arrangement of 10 subunits, each subunit ranging from 350 to 450 kDa, and includes eight globular folded domains known as “functional units”, that are self-assembled into hollow cylinders [29, 30]. While arthropod hemocyanin compared with mollusk hemocyanin are profoundly different in their molecular structure, size, and subunit organization. Arthropod hemocyanins consist of multiples of hexamers, with each hexamer made of monomers of about 75 kDa. For *L*. *vannamei*, its hemocyanin contains 2 subunits of 77 kDa and 75 kDa respectively [19, 31]. Interestingly, our previous studies using the arthropod *L*. *vannamie* demonstrated that hemocyanin from this species has anti-proliferative effects in HeLa cells [32]. However, how arthropod hemocyanin activates the immune system and exerts an antitumor effect *in vivo* has not been evaluated.

To gain a better insight into the antitumor effects of hemocyanin from *L*. *vannamei*, we investigated its effects in an S180 tumor-bearing mouse model. The results showed that hemocyanin exhibited a potent antitumor activity. Furthermore, we believe that the underlying mechanism is dependent on hemocyanin’s ability to enhance immunity and anti-oxidative activity. These results may provide the basis for developing hemocyanin and hemocyanin mimics into antitumor drugs or therapeutic agents for cancer.

## Materials and methods

### Experimental animals and preparation of shrimp sera

Adult Shrimps (*L*.*vannamei***)**, length about 8–12 cm, were purchased from Shantou Huaxun Aquatic Product Corporation (Shantou, Guangdong, China) and maintained in 25 L open-circuit filtered seawater tanks at room temperature with aeration. Shrimps were acclimatized to laboratory conditions for 2 days before experiments.

S180 tumor-bearing Kunming mice (20–25 g), half male and half female, were supplied by the Experimental Animal Center, Xiamen University (Xiamen, China). The animals were kept under standard laboratory conditions with free access to food and tap water, constant room temperature of 25±2°Cand a humidity of 50–60% under a natural day-night cycle.

Hemolymph was drawn directly from *L*.*vannamei* pericardial sinus using a sterile syringe and needle, sera were separated as previously described [17] and stored at -20°Cuntil analysis. The study protocol was approved by the Institutional Animal Care and Use Committee of Shantou University.

### Purification and identification of hemocyanin

Hemocyanin purification was performed by affinity chromatography as previously described with modification [19]. Briefly, a ligand of rabbit anti-shrimp hemocyanin subunit around 75 kDa antibody was allowed to bind an affinity chromatography column according to conventional methods. *L*. *vannamei* sera (200 μl) were loaded onto the affinity column, the column was washed with PBS (0.01 M, pH 7.4) until the absorbance at 280 nm reached baseline. Bound protein (potential hemocyanin) was eluted with glycine-HCl buffer (0.1 M, pH 2.4) and neutralized immediately with Tris–HCl buffer (1 M, pH 8.0). After elution of the protein from the column, the protein was concentrated using Amicon Ultra-15 centrifuge filter units, during which sterilized 0.01M pH7.4 PBS was added to the filter units and centrifuged so as to remove any impurities. The total protein concentration was determined by the Bicinchoninic Acid assay (Genstar, China), and concentrated protein which now contains the sterilized 0.01M pH7.4 PBS was stored at -20°C for subsequent experiments. The whole process was under sterile and pyrogen-free conditions. Prior to use, the stored hemocyanin is allowed to thaw completely and centrifuged at 20000 g for 30 min to sediment any particulates before injection. The purified hemocyanin was validated by gel electrophoresis and immunoblotting assays. SDS polyacrylamide gel electrophoresis (SDS-PAGE) was carried out under reducing conditions on a 10% polyacrylamide separating gel with a 5% polyacrylamide stacking gel. The gel was stained with Coomassie Brilliant Blue R-250. Following SDS-PAGE, immunoblotting assays were further performed. The proteins were transferred to a PVDF membrane with a semi-dry transfer apparatus (LIUYI DYCP-40C, China). The membrane was blocked for 1 h with 5% skim milk in TBST (20 mM Tris, 0.15 M NaCl, 0.1% Tween-20, pH 7.4) at room temperature, then incubated with rabbit anti-shrimp hemocyanin antisera (1:1500 dilution) and goat anti-rabbit IgG-HRP (1:3000 dilution) at room temperature for 1 h and 40 min respectively. Finally, the membrane was washed and developed with substrate (3'3-diminobenzidine, DAB).

### Cell culture

HeLa cells (Cervical carcinoma line), a kind gift from the Research Institute for Biomedical and Advanced Materials, Shantou University, Shantou 515063, China, were grown in Dulbecco’s Modified Eagle’s medium (DMEM, Thermo, USA) supplemented with 10% fetal bovine serum (FBS, Gibco, USA) and 1% penicillin/streptomycin and kept in a 5% CO_2_ incubator at 37°C.

### Mice treatment and grouping

A total of 42 Kunming mice; half male and half female were used. Firstly, 3 male and 3 female mice were randomly selected as normal group, while the rest of the mice were inoculation with the tumor cells by the method described by Pan *et al*. [33]. Briefly, the mice were injected subcutaneously via their armpits with 200 μl of mouse ascitic tumor cells (1.4×10^7^/ml). The tumor cells injection was carried out by the experimental animal center of Xiamen University. After 24 h, all the tumor-injected mice were randomly grouped into 6 groups (equal number of male and female mice with 6 per each group) including 3 hemocyanin treatment groups [high dose group (H), medium dose group (M) and low dose group (L)] and 3 control groups [model group, Fluorouracil (5-FU) group and bovine serum albumin (BSA) group]. For the hemocyanin group, hemocyanin was diluted in sterile normal saline (0.9%) to the required concentrations based on each mouse’s daily weight and 200 μl used for each injection [i.e., 6 mg·kg^-1^bw·d^-1^, hemocyanin (H), 4 mg·kg^-1^bw·d^-1^, hemocyanin (M) and 2 mg·kg^-1^bw·d^-1^, hemocyanin (L)]. For the controls, 200 μl/day of sterile normal saline (0.9%) was injected into the normal and model groups, 6 mg·kg^-1^bw·d^-1^ of 5-FU was injected into the 5-FU group, while 4 mg·kg^-1^bw·d^-1^ of BSA (Sigma, USA) was injected into the BSA group. All the mice were intraperitoneally injected daily for 8 days. Animals were observed daily for activity, growth, fur loss or appearance, feeding, and any abnormal behavior.

### Assessment of tumor weight, size and indices of thymus, liver and spleen

Twenty-four hours after the last treatment administration on day 8, the mice were sacrificed, and the liver, spleen, thymus and solid tumors were removed and weighed. The antitumor activity *in vivo* was expressed as an inhibitory rate calculated by the formula (%): [(A–B)/A] × 100%, where, A and B were the mean tumor weights of the model control and experimental groups respectively. The tumor size was measured using calipers. Tumor volume was estimated as width^2^ × length × 0.52. The liver, spleen and thymus were evaluated by the organ index formula (mg/10 g): liver, spleen or thymus weight (mg)/body weight (10 g).

### Histological investigation

The tumor specimens were fixed in 10% buffered formalin solution for 12 h, followed by dehydration in 70%, 80%, 90%, 95%, and 100% ethanol for 2 h each time, then infiltrated with xylene and paraffin and embedded in paraplast. The specimens were serially sectioned (4 μm thickness) and stained with haematoxylin-eosin (H&E). The slides were observed for histopathological changes and microphotographs were taken using an Olympus BX50 microscope system (Olympus, Japan).

### Splenic lymphocytes proliferation assay

A suspension of spleen cells was diluted to 2×10^6^ cell/ml, 200 μl of the cell suspension was seeded into 96-well plates, 4 wells per each group (i.e., normal group, model group, 5-FU group, BSA group and hemocyanin group), three of them with 5 μl of 200 mg/ml ConA (final concentration 5 μg/ml) and one well set as blank control with 5 μl of the same complete culture medium. The plates were incubated for 68 h in a 5% CO_2_ incubator at 37°C. After the incubation, 100 μl of cell media from each well was taken and discarded, then 20 μl of MTT reagent (5 mg/ml) was added to each well and the plates were incubated for another 4 h. Following this, the culture medium was discarded and 120 μl DMSO (37°C) added to each well. The absorbance was measured using a microplate reader (ELX800, BioTek) with the detection wavelength set at 570 nm and the reference wavelength at 630 nm. The proliferation capacity of spleen lymphocytes was analyzed using the MTT assay [34].

$$\text{Lymphocyte proliferation capacity }\left(\%\right)=\left({\text{OD}}_{\text{ConA }}{}_{\text{treated group}}–{\text{OD}}_{\text{control group}}\right)$$

### Cytotoxic activity of natural killer (NK) cells

The procedure used to detect the capacity of natural killing in NK cells was performed according to the method of Fátima *et al*. [35]. Spleen cells, obtained by mechanical disruption of spleens in sterile conditions as the effector cells, were diluted to 2×10^7^/ml with complete culture medium. HeLa cells were prepared as the target cell and were diluted to 2×10^5^/ml with complete culture medium. 100 μl of the target cell suspension was pipetted into 96-well plates with 4 wells per each group (i.e., normal group, model group, 5-FU group, BSA group and hemocyanin group) and incubated for 8 h. The isopycnic effector cells were added into three wells per group, adjusting the ratio of effector cells to target cells to 100:1. The remaining well was set as target cell control while another well with spleen cells was used as the effector cell control. The cells were incubated in a 5% CO_2_ incubator at 37°C for 4 h. After this, 100 μl of cell media from each well was discarded, then 20 μl of MTT reagent (5 mg/ml) was added to each well and the plates were incubated for 3 h. Following this, the culture medium was removed and 120 μl DMSO (37°C) added to each well. The absorbance was measured using a microplate reader (ELX800, BioTek) with the detection wavelength set at 570 nm and the reference wavelength at 630 nm. Cell survival rate was determined by the MTT assay using the following formula:

$$\text{Natural killing activity of NK cells }\left(\%\right)=\left[{\text{OD}}_{\text{target cell group}}-\;\left({\text{OD}}_{\text{target cell treated with effector cell group}}-{\text{OD}}_{\text{effector cell group}}\right)\right]\;/{\text{ OD}}_{\text{target cell group}}$$

### Determination of serum and liver SOD, MDA and TNF-α levels

Blood was drawn from the orbital venous plexus 24 h after day 8 and immediately transferred into test tubes, and kept at room temperature for 1 h. The blood was then centrifuged at 3500 rpm for 15 min and serum stored at -20°C. The fresh mice liver was washed with cold normal saline, dried with sterile filter paper, weighed, and homogenized in nine volumes of normal saline on ice. The tissue homogenate (10%) was centrifuged at 4000 rpm for 13 min at 4°C and the supernatant was stored at -20°C until analysis.

The serum and 0.25% liver homogenates superoxide dismutase (SOD) were measured by an assay kit (Nanjing Jiancheng Corp, China), the serum and 10% liver homogenates malondialdehyde (MDA) were detected with an analysis kit (Beijing Beyotime Corp, China), and the serum tumor necrosis factor-alpha (TNF-α) were measured by an assay kit (Wuhan Boster Corp, China) according to the manufacturers’ instructions respectively.

### Statistical analysis

Data were expressed as mean ± SEM or mean ± SD for at least three experiments. Student’s t-test was used to compare data. A p-value less than 0.05 was considered statistically significant.

## Results

### Purification and identification of hemocyanin

**Fig 1:**
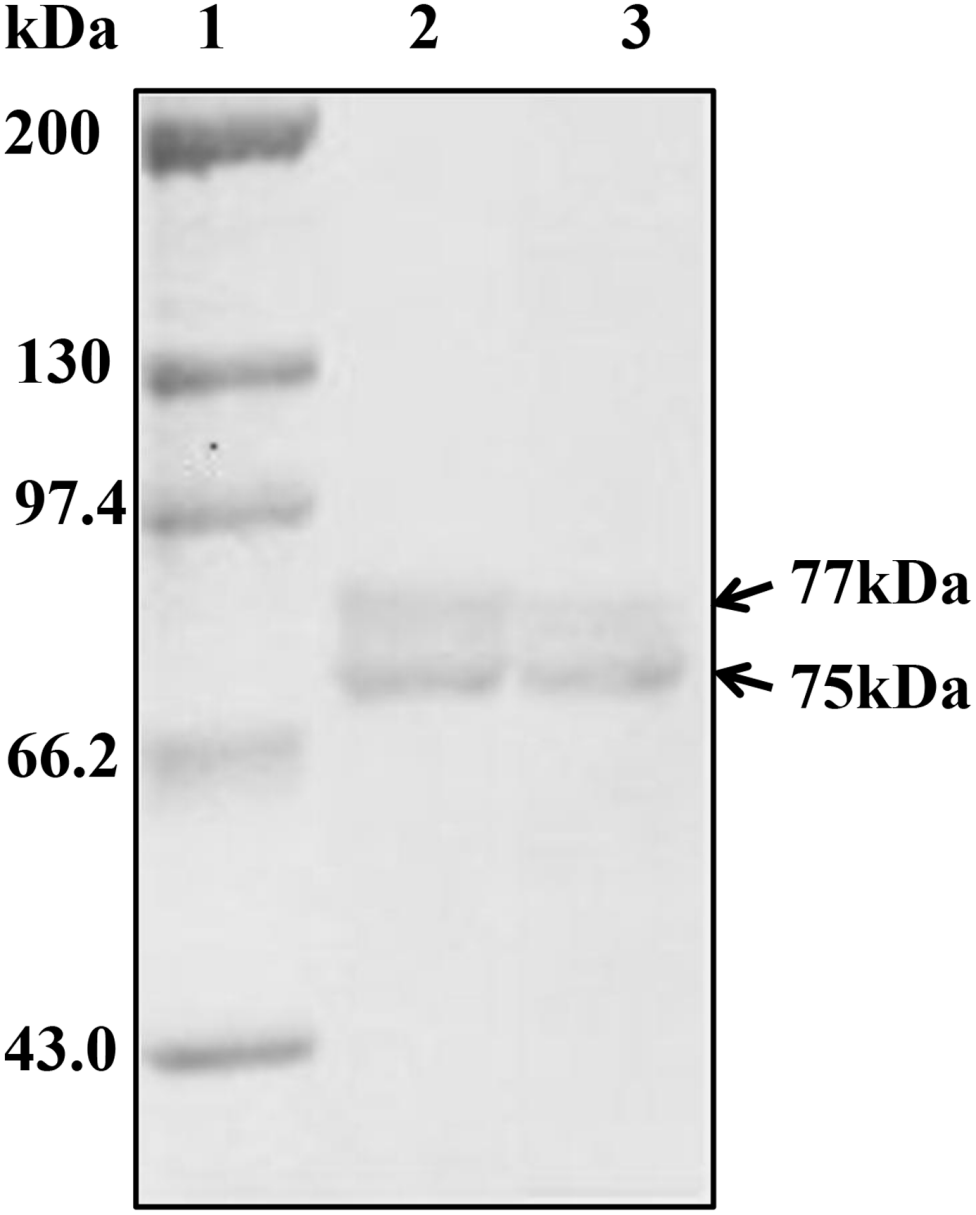
SDS-PAGE and Western-blotting analysis of purified hemocyanins. 1: molecular mass markers; 2: SDS-PAGE analysis of hemocyanin; 3: hemocyanin incubated with rabbit anti-hemocyanin antibodies (1:1500) and goat anti-rabbit IgG-HRP (1:3000) for immunoblotting.

Hemocyanin was purified from *L*. *vannamei* heamolymph by affinity chromatography. The purity and molecular weight of the eluted protein was analyzed by gel electrophoresis. As shown in Fig 1, two bands about 75 and 77 kDa were observed by SDS-PAGE. Furthermore, the protein bands reacted specifically with anti-shrimp hemocyanin antibody (Fig 1). The results confirmed that a good hemocyanin purification had been achieved.

### Hemocyanin possess antitumor activities on S180 tumor-bearing mice

**Fig 2:**
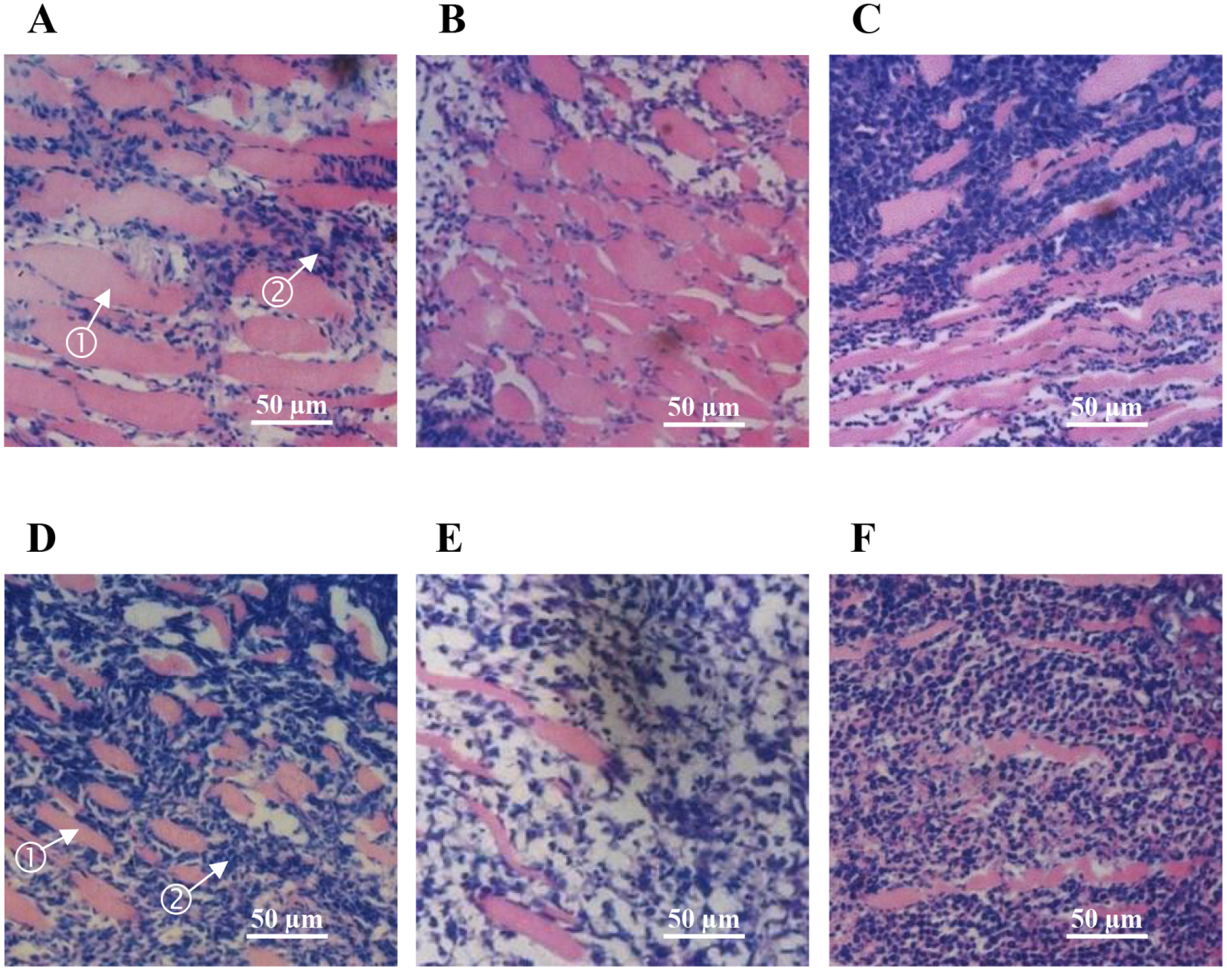
Histopathology and cytopathology section of the S180 tumor-bearing mice tumor tissue stained with H&E (200X). (A): Hemocyanin (H) group (6 mg·kg^-1^bw·d^-1^). (B): Hemocyanin (M) group (4 mg·kg^-1^bw·d^-1^). (C): Hemocyanin (L) group (2 mg·kg^-1^bw·d^-1^). (D): 5-FU group (6 mg·kg^-1^bw·d^-1^). (E): Model group. (F): BSA group (4 mg·kg^-1^bw·d^-1^). Arrow 1: Fibrotic tumor tissues. Arrow 2: S180 sarcoma cells.

To investigate whether hemocyanin decreased the tumor growth rate *in vivo*, S180 tumor cells were implanted under the skin by injecting the mice to establish a tumor model. As shown in Table 1, hemocyanin exhibited significant antitumor effects on S180 tumor-bearing mice. The tumor inhibition rates in hemocyanin (H), hemocyanin (M) and hemocyanin (L) groups were determined to be 34%, 49% and 24%, respectively. The mean value of tumor weight in hemocyanin (M) group was 50% less than model group (*p* < 0.01), while no measurable differences were detected between BSA group and model group. Similarly, the tumor weight and volume were also decreased significantly (*p*<0.05 or 0.01) in hemocyanin (H) and hemocyanin (M) groups compared with the model group. Additionally, the tumor cell number decreased significantly, while the fibrous tissue increased significantly in the tumor tissues with hemocyanin treatment (Fig 2A, 2B and 2C), especially for hemocyanin (M) group (Fig 2B). On the other hand, in both model group and BSA group there was a significant increase in tumor cells with their nuclei seen as a large dense patch but a decrease in fibrous tissue (Fig 2E and 2F). Therefore, these findings implied shrimp hemocyanin possessed antitumor activities against S180 tumor-bearing mice.

**Table 1:** Effect of hemocyanin from shrimp *Litopenaeus vannamei* on the immune ability of mice bearing S180 sacrcoma.

Group	Dosage (mg·kg^-1^bw·d^-1^)	Tumor weight	Inhibition rate (%)	Tumor volume(cm^3^)	Liver index (mg/10g)	Spleen index (mg/10g)	Thymus index (mg/10g)
Normal	/	/	/	/	768.35±70.61	66.21±20.79	38.28±4
Model	/	0.9±0.05	0	1.19±0.38	670.15±55.57	73.39±20.14	32.70±7.53
5-FU	6	0.66±0.64	27	0.92±0.42	660.57±9.43	69.10±7.26	29.50±6.88^##^
Hemocyanin (H)	6	0.59±0.32*	34	0.83±0.11*	776.29±33.7*	90.49±4.51**	31.77±7.42
Hemocyanin (M)	4	0.46±0.08**	49	0.58±0.11*	753.02±70.07	90.54±8.99**	32.55±5.32
Hemocyanin (L)	2	0.68±0.06	24	1.08±0.3	772.67±38.77*	102.26±21.94*	37.40±4.34
BSA	4	0.89±0.16	1.1	1.87±0.26	679.39±23.5	84.84±1.89	31.40±2.91

The mice were injected intraperitoneally (IP) with hemocyanin [hemocyanin(H) group, 6 mg·kg^-1^bw·d^-1^; hemocyanin(M) group, 4 mg·kg^-1^bw·d^-1^; hemocyanin(L) group, 2 mg·kg^-1^bw·d^-1^] or left untreated (Model) for 8 days. The normal group was not treated with cancer cells or hemocyanin. The 5-FU group was injected with cancer cells without hemocyanin but 5-FU (6 mg·kg^-1^bw·d^-1^). Values are expressed as mean ± SD for 6 mice in each group. Comparison between two groups was down using one-way.

*ANOVA vs Model p<0.05.

**ANOVA vs Model p<0.01.

^##^ANOVA vs Normal p<0.01.

### Effect of hemocyanin on organ index in S180 tumor-bearing mice

As shown in Table 1, compared with the model group, the liver index of hemocyanin(H) and hemocyanin(L) groups increased significantly (p<0.05), while the spleen index of all hemocyanin dose groups increased significantly (p<0.05 or 0.01). The thymus index in the 5-FU group notably decreased (p<0.01) compared with the normal group, which seems to suggest that the thymus had been severely damaged, implying that 5-FU had a harmful effect while hemocyanin does not. It should be noted that all hemocyanin dose groups also had to some degree an increase in organ index compared with the 5-FU group. Therefore, hemocyanin not only exerts antitumor activity but may also display protective effects on liver and spleen.

### Study of splenic lymphocyte proliferation capacity

**Fig 3:**
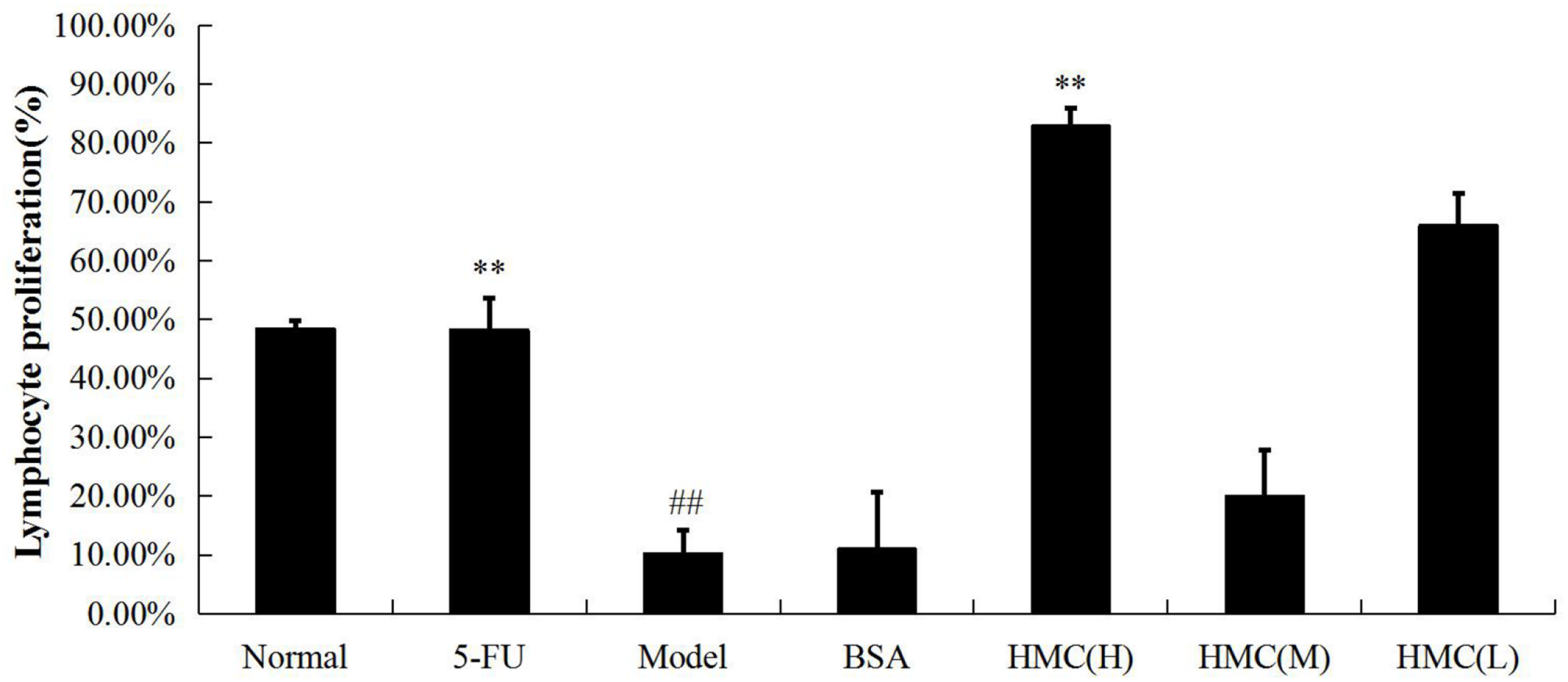
Splenic lymphocyte proliferation capacity of tumor-bearing mice. The mice were injected intraperitoneally (IP) with hemocyanin [hemocyanin (H) group, 6 mg·kg^-1^bw·d^-1^; hemocyanin (M) group, 4 mg·kg^-1^bw·d^-1^; hemocyanin (L) group, 2 mg·kg^-1^bw·d^-1^] or left untreated (Model) for 8 days. The normal group was not treated with cancer cells or hemocyanin. The 5-FU group was injected with cancer cells without hemocyanin but 5-FU (6 mg·kg^-1^bw·d^-1^). Values are means ± SEM **p<0.01 vs. Model; ##p <0.01 vs. Normal.

As shown in Fig 3, the proliferation of spleen lymphocytes of model group decreased significantly (p<0.01) compared to normal group. However, spleen lymphocytes from the hemocyanin (H) group showed nearly 2-fold increase in proliferation than normal group (p<0.01). This result seems to indicate that hemocyanin could improve and enhance the ability of ConA to induce the proliferation of T lymphocytes.

### Evaluation of cytotoxic activity of NK cells

**Fig 4:**
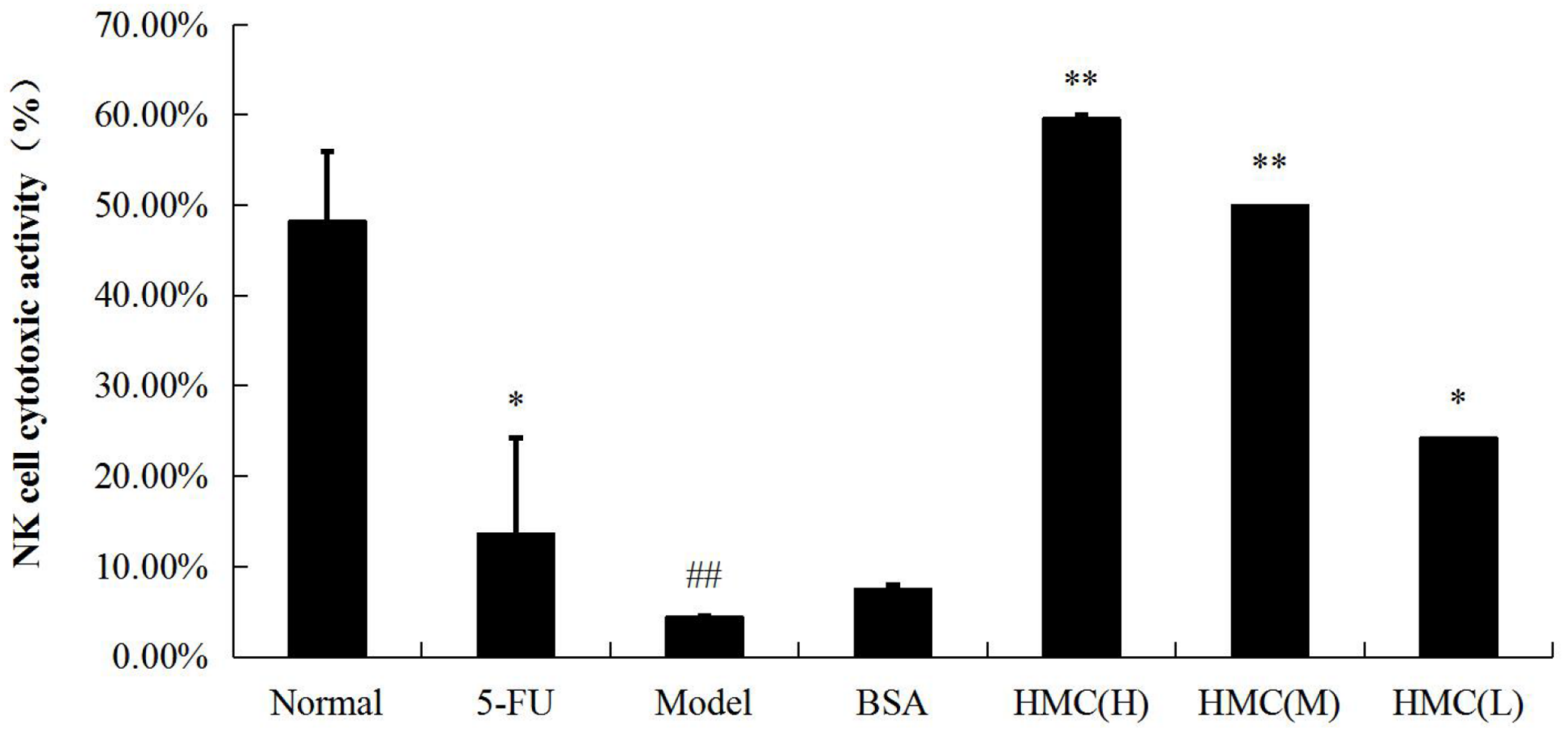
Effect of hemocyanin on cytotoxic activity of NK cells of mice bearing S180. After 8 days treatment, MTT assay was used to determine the cell survival rate at 570 nm. A single asterisk indicates hemocyanin (L), (2 mg·kg^-1^bw·d^-1^) and 5-FU vs Model p<0.05; Double asterisks indicates hemocyanin (H), (6 mg·kg^-1^bw·d^-1^) and hemocyanin (M), (4 mg·kg^-1^bw·d^-1^) vs Model p<0.01; Double # indicates Model vs Normal p<0.01. Data are means ± SEM of three independent experiments.

To understand the consequence of hemocyanin treatment on the innate immune response, we measured NK cell activity on day 8 post-treatment. The effects of hemocyanin on NK cell activity are shown in Fig 4. It can be seen that NK cell activity were significantly lower (p<0.01) in the model than in the normal group. Notably, mice treated with hemocyanin (H), hemocyanin (M), and hemocyanin (L) all had significantly higher NK cell activity than model group (p<0.05 or 0.01).

### Hemocyanin treatment effects on TNF-α

**Fig 5:**
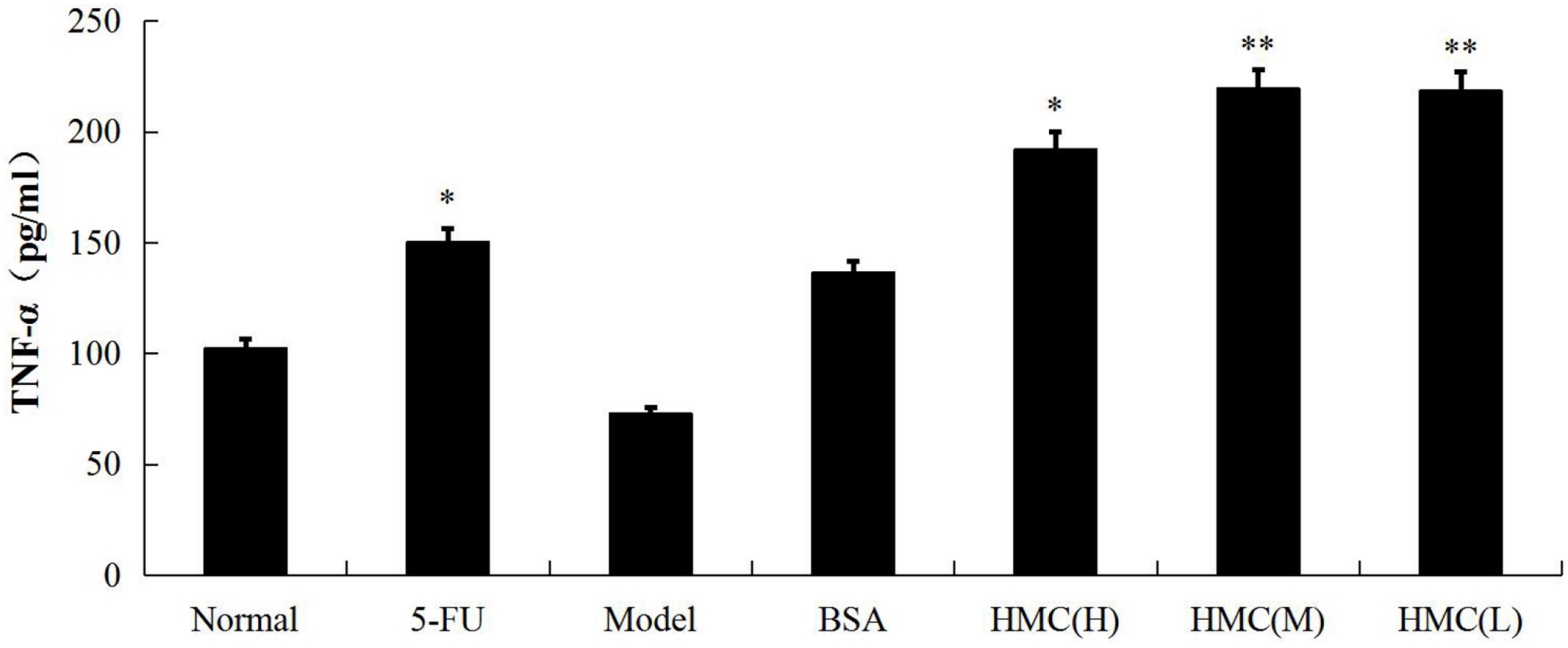
Effect of hemocyanin on serum cytokine TNF-α level. Group of 6 mice treated with hemocyanin or BSA at different concentrations [hemocyanin (H) group, 6 mg·kg^-1^bw·d^-1^; hemocyanin (M) group, 4mg·kg^-1^bw·d^-1^; hemocyanin (L) group, 2mg·kg^-1^bw·d^-1^; BSA group, 4 mg·kg^-1^bw·d^-1^]. On day 8 the serum TNF-α was determined by ELISA. Results show each group compared with Model group. Data are means ± SEM of three independent experiments. (*p<0.05, **p<0.01).

TNF-α concentration in serum was detected using ELISA. As shown in Fig 5, while hemocyanin (H) had significantly higher TNF-α concentration (p<0.05) than model group, there was a marked increase (p<0.01) in the hemocyanin (M) and hemocyanin (L) groups compared to the model group. This shows that hemocyanin can significantly stimulate the secretion of TNF-α after 8 days treatment.

### Determination of SOD and MDA level in serum and liver

In the serum and liver of the model group, there was a significant decrease (p<0.01) in the activities of SOD compared with the normal group, while the content of MDA significantly increased (p<0.01) in the model group (Table 2). Interestingly, we found that hemocyanin (M) treatment significantly inhibited MDA generation (p<0.01) which significantly improved SOD enzyme activity (p<0.01) in serum. Mice treated with hemocyanin (H), hemocyanin (M) and hemocyanin (L) could all elevate the activities of SOD and reduce the level of MDA in liver, with the most significant (p<0.01) change observed in the hemocyanin (H) group.

**Table 2:** Effect of Hemocyanin from Shrimp *Litopenaeus vannamei* on the SOD activities and MDA level of mice bearing S180 sacrcoma.

Group	Dosage (mg·kg^-1^bw·d^-1^)	serum		liver	
		SOD (U/mL)	MDA (mmol/mL)	SOD (U/mgprot)	MDA (nmol/mgprot)
Normal	/	71.33±3.03	13.67±4.51	335.75±33.27	7.4±0.005
Model	/	51.76±18.42^##^	17.51±4.37^##^	171.06±72.34^##^	14.35±0.167^##^
5-FU	6	48.55±8.71	15.61±3.17	265.25±25.51	11.13±0.005
Hemocyanin(H)	6	69.30±19.43*	17.18±2.43	489.31±41.01**	6.95±1.33**
Hemocyanin(M)	4	89.86±11.59**	12.79±2.76**^#^	413.37±53.82**	8.37±1.25*
Hemocyanin(L)	2	50.70±7.3	12.77±1.80**^#^	219.46±58.74	8.04±1.42*
BSA	4	54.65±10.34	21.03±3.19	114.87±21.71	15.12±0.84^#^

The mice were injected intraperitoneally (IP) with hemocyanin [hemocyanin (H) group, 6 mg·kg^-1^bw·d^-1^; hemocyanin (M) group, 4 mg·kg^-1^bw·d^-1^; hemocyanin (L) group, 2 mg·kg^-1^bw·d^-1^] or left untreated (Model) for 8 days. The normal group was not treated with cancer cells or hemocyanin. The 5-FU group was injected with cancer cells without hemocyanin but 5-FU (6 mg·kg^-1^bw·d^-1^). Values are expressed as mean ± SD for 6 mice in each group. Comparison between two groups was down using one-way.

*ANOVA vs Model p<0.05.

**ANOVA vs Model p<0.01.

^#^ANOVA vs Normal p<0.05.

^##^ANOVA vs Normal p<0.01.

## Discussion

Several previous research findings have shown that hemocyanin is a multifunctional protein [1–6]. Most importantly, it has been demonstrated that hemocyanin from mollusk is endowed with a significant antitumor effect [36–39]. Similarly, we previously reported that the shrimp *L*. *vannamei* hemocyanin also displays obvious antiproliferative effect against HeLa cells [32], but it’s antitumor properties *in vivo* are unknown. S180 tumor-bearing mice are a common tumor model for preliminary screening and evaluation of antitumor drugs [40, 41]. Therefore, in this study, we used S180 tumor-bearing mice as an animal model to investigate the antitumor function of hemocyanin from *L*. *vannamei*. Here, we found that hemocyanin exhibits an obvious antitumor activity (Table 1 and Fig 2).

In antitumor immunity, which is predominantly cellular, T cells are the main immune cells that can exert direct cytotoxicity or indirectly through the secretion of cytokines, TNF-α, and IFN-γ. Many of the cytokines such as IFN-γ or IL-2 can activate natural killer (NK) cells [41], which play a fundamental role in immune surveillance and coordinate responses of other immune cells [42]. Previously, it was reported that CCH is able to enhance T helper type 1 immunity, increases NK cell activity and stimulates INF-γ secretion to prevent tumor growth in a murine bladder cancer model [24]. Recently, in a murine model of sarcoma cells, KLH was found to increase the antitumor activity of NK cells *in vitro* [43]. The above reports support our results in which we demonstrated that hemocyanin from shrimp *L*.*vannamei* could promote ConA to induce the proliferation and transformation of T-lymphocytes in hemocyanin treated S180 tumor-bearing mice (Fig 3). Similarly, we found that hemocyanin improves the cytotoxic activity of NK cells in S180 tumor-bearing mice (Fig 4).

TNF-α has various antitumor functions including inducing apoptosis, affecting the tumor vascular system and immune regulation [44]. Meanwhile, TNF-α can augmented cellular maturation of dendritic cells and induce more robust T-cell activation [45]. Recent studies have confirmed that hemocyanin is internalized by macrophages through pinocytic vesicles and by clathrin-dependent endocytosis, and that it activates posttranscriptional mechanism of macrophages and thus potentiates TNF-α protein secretion [46]. In line with this, we demonstrated a significant enhancement of TNF-α secretion in S180 tumor-bearing mice after 8 d treatment with hemocyanin (Fig 5).

The immune organs index can directly reflect the level of immune functions of the body, and the effects of drugs on these organs can be used as the preliminary indicator for the study on immuno-pharmacological mechanisms in animals [47]. Interestingly, given that hemocyanin treatment significantly increased the organ indices, it means hemocyanin does not have any adverse effects, and therefore offers some protection of these organs (Table 1). Collectively, these results have revealed that hemocyanin improves immunity.

Oxidative stress and oxidants such as reactive oxygen species (ROS) and MDA are involved in carcinogenesis by inducing mutations [48]. MDA is considered a biomarker of oxidative stress [49], while SOD is an antioxidant which protects animals against ROS. In the present study, hemocyanin was able to augment the activity of SOD as well as significantly decrease the level of MDA (Table 2). These observations were in accordance with other previous reports [33, 50], indicating that hemocyanin can exert antitumor effects as well as improve immunoregulatory activity via its antioxidant properties.

Interestingly, while there was an antitumor effect by the three doses of hemocyanin tested, there was no clear-cut dose-effect relationship (Figs 3 and 5, Tables 1 and 2). In most cases, hemocyanin (M) was most effective than both hemocyanin (H) and hemocyanin (L). This observation could probably be due to the fact that there might be no threshold in response, but with the medium dose eliciting the optimum response. Besides this, as a foreign protein, hemocyanin could activate both innate and adaptive immune response when injected into mice as an immunostimulant. At the same time, it has previously been reported to have anti-proliferation effects on mammalian cells [32]. Therefore these complex effects and multifunctionality of hemocyanin could probably explain the no clear-cut dose-effect relationship observed. A similar phenomenon has been observed in S180 tumor-bearing mice stimulated with abnormal Savda Munziq [41].

In conclusion, the present study shows that hemocyanin from *L*. *vannamei* could exert an antitumor effect in S180 tumor-bearing mice. The observed antitumor effect induced by hemocyanin might be due to the immunity enhancement and anti-oxidative activity. However, in order to substantiate these findings, further work using additional tumor models like the nude mice model will have to be used.

## Supporting information

S1 File ARRIVE checklist. (DOCX)
