# Supplementary material for: Litopenaeus vannamei hemocyanin exhibits antitumor activity in S180 mouse model in vivo
Source: PLoS One. 2017 Aug 30;12(8):e0183783. doi: 10.1371/journal.pone.0183783 (PMC5576664; doi:10.1371/journal.pone.0183783)

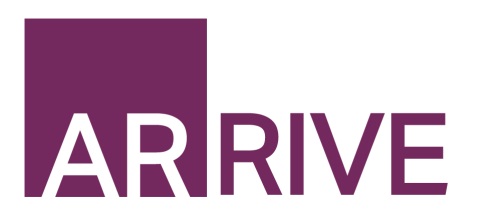


The ARRIVE Guidelines Checklist

Animal Research: Reporting In Vivo Experiments

Carol Kilkenny^1^, William J Browne^2^, Innes C Cuthill^3^, Michael Emerson^4^ and Douglas G Altman^5^

*^1^The National Centre for the Replacement, Refinement and Reduction of Animals in Research, London, UK, ^2^School of Veterinary Science, University of Bristol, Bristol, UK, ^3^School of Biological Sciences, University of Bristol, Bristol, UK, ^4^National Heart and Lung Institute, Imperial College London, UK, ^5^Centre for Statistics in Medicine, University of Oxford, Oxford, UK.*

|  | | ITEM | RECOMMENDATION | Section/ Paragraph |
| --- | --- | --- | --- | --- |
| 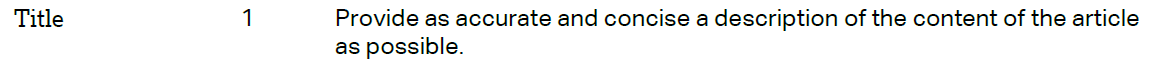 | | | Title page, top line |  |
| 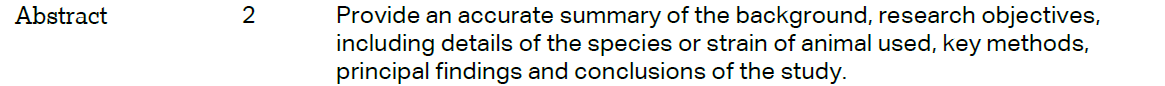 | | | Abstract, page 2 |  |
| INTRODUCTION | | |  |  |
| 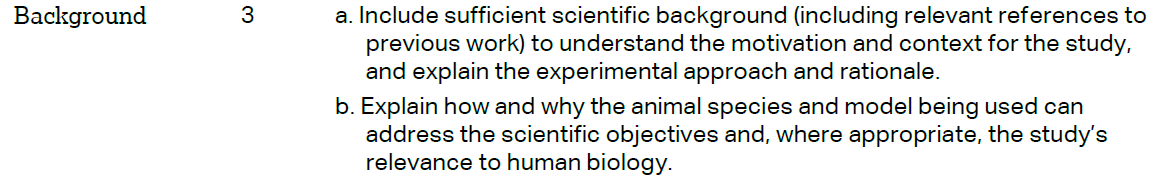 | | | a. Introduction  Para 1, 2& 3  b. Discussion  Para 1 |  |
| 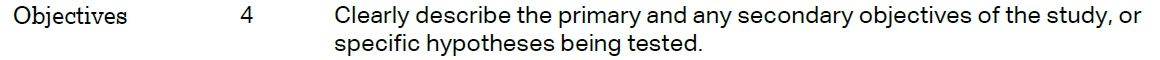 | | | Introduction  Para 4 |  |
| METHODS | | |  |  |
| 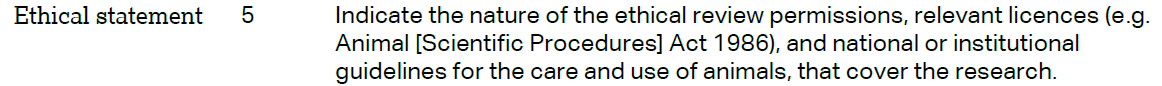 | | | Methods  Para 3 |  |
| 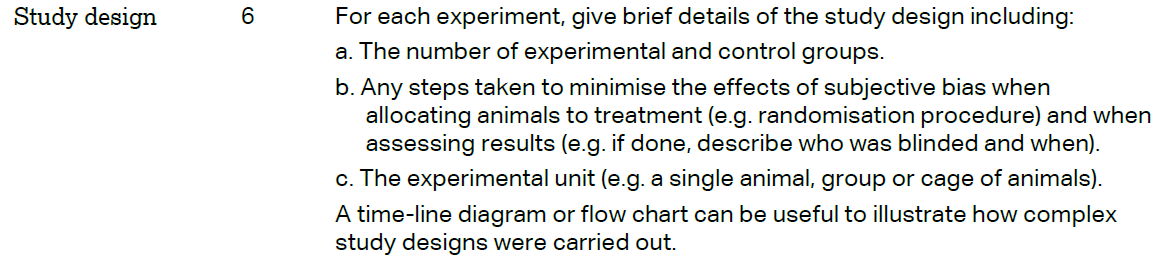 | | | Methods  a. Para 6  b. Para 6  c. Para 6 |  |
| 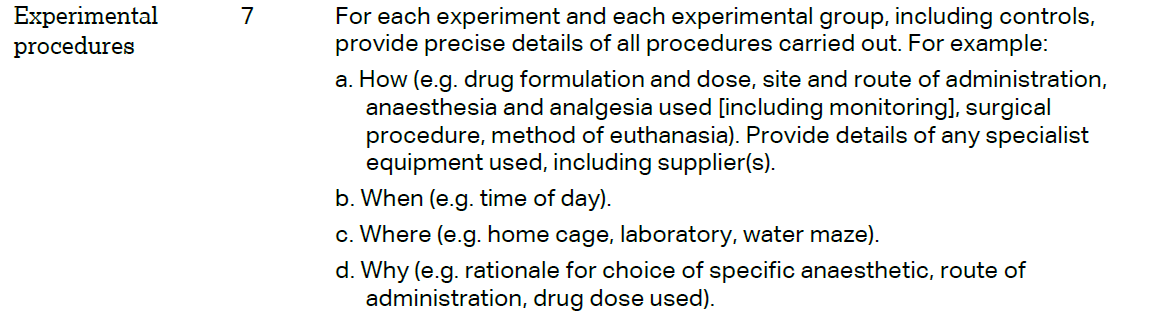 | | | Methods  a. Para 6  b. Para 6  c. Para 2  d. Para 6 |  |
| 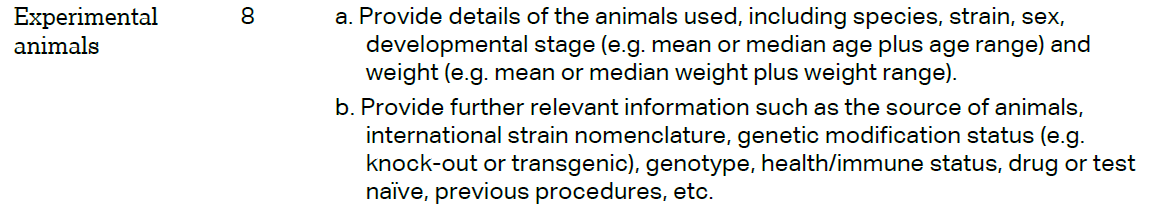 | | | Methods  a. Para 2  b. Para 2 |  |

The ARRIVE guidelines. Originally published in *PLoS Biology*, June 2010^1^

| 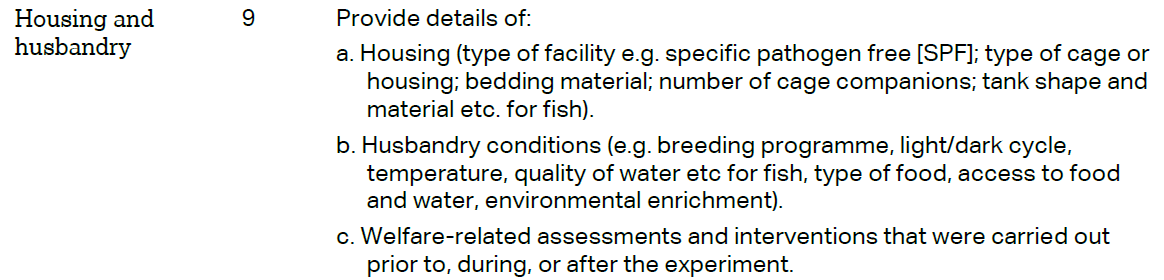 | Methods  a. Para 2  b. Para 2  c. Para 6 |  |
| --- | --- | --- |
| 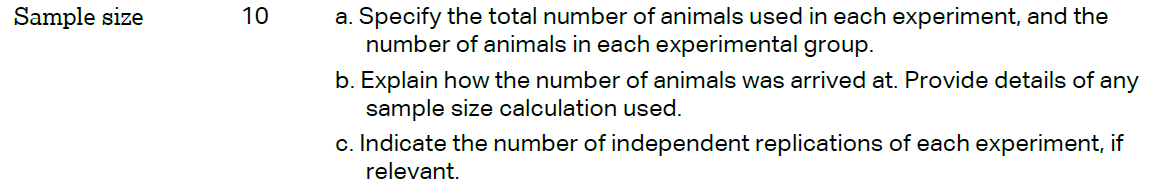 | Methods  a. Para 6  b. Para 6  c. Para 13 |  |
| 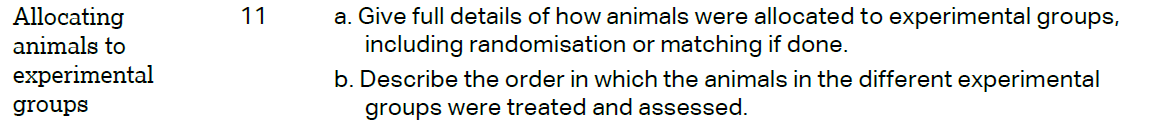 | Methods  a. Para 6  b. Para 6 |  |
| 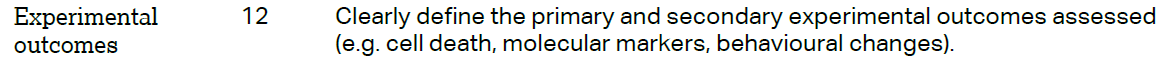 | Results  Para 2 |  |
| 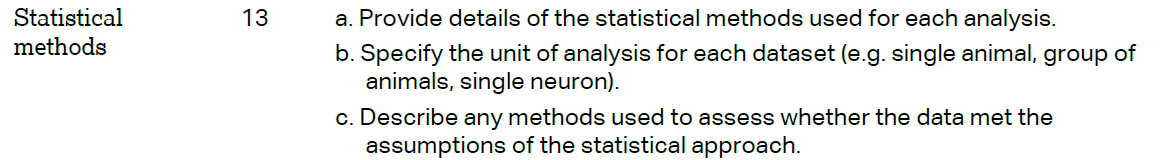 | Methods  a. Para 13  b. Para 13  c. Para 13 |  |
| RESULTS |  |  |
| 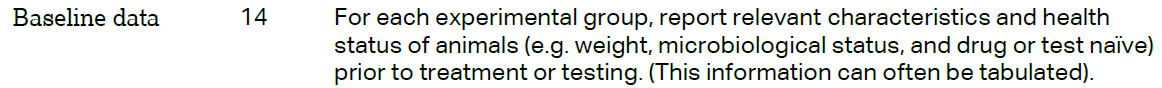 | Methods  Para 6 |  |
| 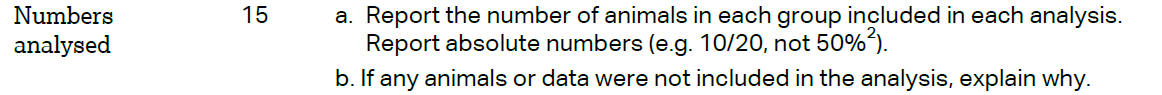 | a. Methods, Para 6  b. Results  Table 1&2 legends |  |
| 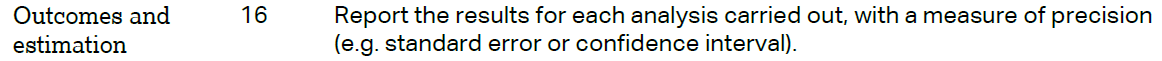 | Methods, Para 13  Figure legends 3, 4 & 5 |  |
| 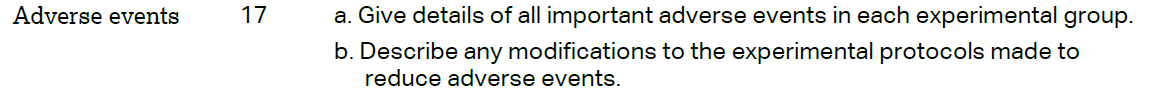 | N/A |  |
| DISCUSSION |  |  |
| 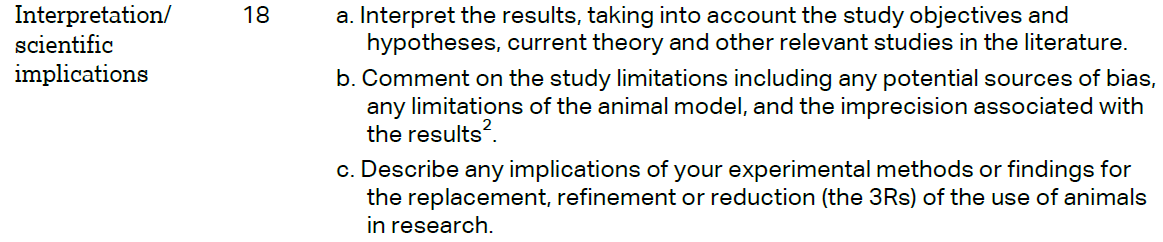 | Discussion  a. Para 1& 2  b. Para 7  c. N/A |  |
| 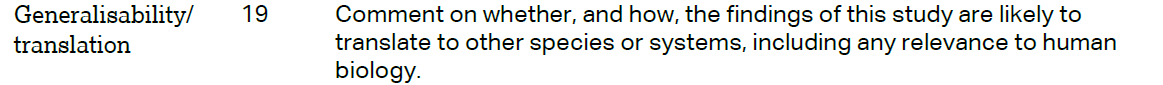 | Introduction,  Para 4 |  |
| 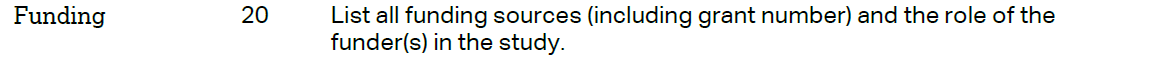 | | Funding, Para 1 |


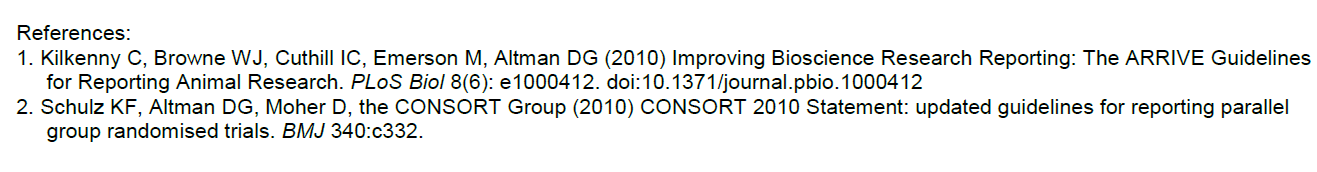

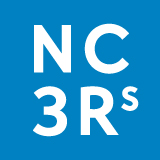

Supplement: S1 File — (DOCX) [file pone.0183783.s001.docx]
